# The Dublin Dissector, or Manual of Anatomy; Comprising a Description of the Bones, Muscles, Vessels, Nerves, and Viscera; Also, the Relative Anatomy of the Different Regions of the Human Body, Together with the Elements of Pathology

**Published:** 1840-10-31

**Authors:** 


### MEDICAL EXAMINER.

DEVOTED TO MEDICINE, SURGERY, AND THE COLLATERAL SCIENCES.

No. 44.] PHILADELPHIA, SATURDAY, OCTOBER 31, 1840. [Vol. III.

### BIBLIOGRAPHICAL NOTICE.

*The Dublin Dissector, or Manual of Anatomy; comprising a Description of the Bones, Muscles, Vessels, Nerves, and Viscera; also, the Relative Anatomy of the different regions of the Human Body, together with the Elements of Pathology.* By Robert Harrison, A.M. M.B.T.C.D., Member, and one of the Professors of Anatomy in the Royal College of Surgeons in Ireland, &c. First American, from the fifth enlarged Dublin edition. With additions by Robert Watts, Jr., M.D., Professor of Anatomy in the College of Physicians and Surgeons in the City of New York, &c. &c. New York: J. & H. G. Langley. Philadelphia: Haswell, Barrington & Haswell. 1840.

The emission of a fifth edition is at once proof of the popularity of this work among those who cultivate practical anatomy; and we are happy in being able, from personal experience of its assistance, to endorse the opinion generally entertained of its merits. It is really a superior work;—concise, yet containing all that is necessary,—arranged as a manual for the dissector, and yet embodying principles of pathology, where these may be predicated upon the position and functions of parts, or where they may serve as aids to the memory of the student: but never, by useless digressions, losing sight of the main object of a work of this nature, which should be to enable the student to gain the greatest amount of instruction from each subject, assist him in the choice of the regions upon which he should commence his dissections—to guide him in his incisions, in order that he may not be led into any unnecessary mutilation of parts—and to describe each part in its order of succession as briefly as is consistent with its due comprehension.

The plan of the work exhibits a judicious combination of these several modes of describing special anatomy. It is topographical, for it takes up region after region in the order which contributes most to economise the subject. It follows the order of superposition, for it traces each of the objects in each region, layer by layer, and it is more or less surgical in proportion to the importance of the region and the number of surgical operations required in it; and the consideration of each region is preceded by a concise, yet clear description of the best method of displaying the different organs contained in it.

The reputation of the Dublin Dissector is so completely established that it would be supererogatory to review in detail its fifth edition; but there are some points, exhibiting a close personal examination of the parts described, which it would be improper not to mention. Among these we may cite the arrangement and uses of the crucial ligaments of the knee-joint—conceded by all recent authors, among whom we may cite Professors Horner, Cruveilhier, and Blandin, as limiting extension and rotation inwards, but as being relaxed in flexion of the leg upon the thigh. Cruveilhier even does not give them so extended a function. He says: "Their obliquity and their approach to the posterior part of the articulation render them capable of limiting extension, during which act their points of insertion are separated, while they are approximated during flexion." Blandin assumes, that "the crucial ligaments, being placed nearer the direction of flexion than of extension, serve to limit the latter." The idea of their limiting forced flexion appears not to have occurred to any one; and yet the fact, which is demonstrable, has evidently been caught by the author of the Dublin Dissector, although not enlarged upon. He states that, "The crucial ligaments serve to attach the femur to the tibia; they steady the one bone upon the other, and strengthen the back part of the joint, in the same manner as the patella does in front; they also tend to prevent any lateral displacement: they are both made tense, and of course prevent too much rotation of the tibia inwards, and they are relaxed and separate somewhat in rotation outwards. In extension, also, they are both very tense, especially the posterior. *In flexion the anterior is so, but in a less degree.* We cite this statement as exhibiting the close attention of the author to the minutiæ of anatomy, although the work is only a manual for the dissector; and in connection with it cannot refrain from quoting an extract from a private letter lately received from Professor Chassaignac, one of the most eminent anatomists in Paris, who not only accords the limit to flexion as a function of the anterior crucial ligament, but has also drawn some important deductions from this property. He writes: "I communicate a slight anatomical discovery which I have lately made. You know that all anatomists have described the crucial ligaments as limiting extension. I find that they are wrong: there is but one which has this property; the other limits flexion, and is relaxed in proportion to the degree to which extension is carried: by severing them alternately the exactitude of this fact is demonstrated, and we can recognise that one is a *counter-extensor*—the other, a *counter-flexor*; the latter is the external or anterior. I have already deduced some pathological considerations from this fact. Patients are found capable of assigning no other known cause for a disease of the knee than an excess of flexion. Now, as it is supposed that flexion of the knee has no other limit than the encounter of the two bony surfaces, it is impossible to believe in a simple sprain of the knee by excess of flexion, and yet nothing is more certain. To resume: an anatomical error leads to a physiological error in inducing us to believe that there is no ligamentous limit to flexion of the knee, and this in its turn to a pathological error, leading us to exclude as a cause of disease of the joint its exaggerated flexion."

But to return to the Dublin Dissector: the value of the present edition is certainly enhanced by the notes of Dr. Watts, particularly the ones which collect the muscles into groups, and bring all those of each region under the eye of the student at once, and those which connect with the description of each bone the names of their respective muscles. The rest are not so important. Some, indeed, are merely repetitions of the text in different language; while all have the common fault of being entirely too surgical in their character for the nature of the work, and of referring too frequently to the specimens of private cabinets: one, in fact, occupying the whole of p. 490, is an excellent treatise on inflammation of the joints and its terminations. These add to the size of the volume, without furthering its object—instruction in *practical* anatomy—in a proportionate degree.

The publishers have, we see, added an appendix, much wanted in the original work, containing the best methods of making injections and dried preparations.

On the whole, it is a work which we cordially recommend to the student of practical anatomy.

